# What's in a name; Genetic structure in *Solanum *section *Petota *studied using population-genetic tools

**DOI:** 10.1186/1471-2148-11-42

**Published:** 2011-02-10

**Authors:** Mirjam MJ Jacobs, Marinus JM Smulders, Ronald G van den Berg, Ben Vosman

**Affiliations:** 1 Wageningen UR Plant Breeding, Wageningen University and Research Centre, Droevendaalsesteeg 1, 6708PB Wageningen, The Netherlands; 2 Biosystematics, Wageningen University and Research Centre, Wageningen, The Netherlands; 3 Centre for BioSystems Genomics, P.O. Box 98, 6700 AB Wageningen, The Netherlands

## Abstract

**Background:**

The taxonomy and systematic relationships among species of *Solanum *section *Petota *are complicated and the section seems overclassified. Many of the presumed (sub)species from South America are very similar and they are able to exchange genetic material. We applied a population genetic approach to evaluate support for subgroups within this material, using AFLP data. Our approach is based on the following assumptions: (i) accessions that may exchange genetic material can be analyzed as if they are part of one gene pool, and (ii) genetic differentiation among species is expected to be higher than within species.

**Results:**

A dataset of 566 South-American accessions (encompassing 89 species and subspecies) was analyzed in two steps. First, with the program STRUCTURE 2.2 in an 'unsupervised' procedure, individual accessions were assigned to inferred clusters based on genetic similarity. The results showed that the South American members of section *Petota *could be arranged in 16 clusters of various size and composition. Next, the accessions within the clusters were grouped by maximizing the partitioning of genetic diversity among subgroups (i.e., maximizing Fst values) for all available individuals of the accessions (2767 genotypes). This two-step approach produced an optimal partitioning into 44 groups.

Some of the species clustered as genetically distinct groups, either on their own, or combined with one or more other species. However, accessions of other species were distributed over more than one cluster, and did not form genetically distinct units.

**Conclusions:**

We could not find any support for 43 species (almost half of our dataset). For 28 species some level of support could be found varying from good to weak. For 18 species no conclusions could be drawn as the number of accessions included in our dataset was too low. These molecular data should be combined with data from morphological surveys, with geographical distribution data, and with information from crossing experiments to identify natural units at the species level. However, the data do indicate which taxa or combinations of taxa are clearly supported by a distinct set of molecular marker data, leaving other taxa unsupported. Therefore, the approach taken provides a general method to evaluate the taxonomic system in any species complex for which molecular data are available.

## Background

The taxonomy of wild potato species, belonging to section *Petota *of the genus *Solanum*, is known to be problematic [1-3]. Identification of many species is difficult and the systematic relationships among the wild potatoes are not clear. One of the causes for these difficulties is the ability of many species to hybridize easily [2]. Hawkes [1] hypothesized that approximately 12% of the 224 tuber-bearing *Solanum *species he recognized, had arisen from hybrid speciation. A quote from Correll [4] (page 404) may serve to illustrates the magnitude of the problem: "In fact, the difficulty one encounters in dealing with plants from northwest Argentina and southern Bolivia is such that one is tempted to consider, with very few exceptions the entire Tuberarium population to be one vast assemblage of hybrids" (section *Tuberarium *being roughly equivalent to the current section *Petota*).

Next to hybridization there is a large amount of phenotypic plasticity, i.e., plants look different in different environments [4-6]. Partly because of this, taxonomists have granted minor variants (sub)species status. As a consequence, species boundaries are based on morphological characters that are not expressed under all conditions. Hence, numerous species have been described, many of which are extremely similar to each other, and that is why Spooner and Salas [2] and van den Berg and Jacobs [3] concluded that the group of wild species belonging to *Solanum *section *Petota *is overclassified. An extreme example of overclassification within *Solanum *section *Petota *is the so-called brevicaule complex. Morphological results failed to distinguish the 30 species in the brevicaule complex [7]. Molecular data showed that the brevicaule complex is paraphyletic and that many taxa should probably be relegated to synonymy [8].

The systematic relationships among these species are also hard to determine. These have been expressed in an arrangement of 19 series, as designated by Hawkes [1] and others. Some of the series are difficult to keep apart while other series contain subgroups that could be considered a separate series [3]. To date, the series classification of Hawkes [1] and other authors has received no cladistic support [6]. Jacobs et al. [9] described the taxonomic structure present in *Solanum *section *Petota*. They focused on testing the validity of the series classification and on studying the taxonomic structure of the section based on AFLP data. They produced the largest dataset ever constructed for *Solanum *section *Petota *and analysed it both phenetically and phylogenetically. Although some of the branches in the resulting trees were supported by jackknife values above 69, both phenetic and phylogenetic trees also display a large polytomy containing many taxa.

In the present study, we focus on the status of the recognized species in section *Petota*, in order to evaluate possible overclassification, misclassification and hybridization. The number of species in the *Solanum *section *Petota *has already been reduced somewhat due to the application of molecular techniques. While Hawkes [1] still recognized 227 tuber-bearing species (of which 7 were cultivated) and 9 non tuber-bearing species within section *Petota*, Spooner and Hijmans [5] recognized only 203 tuber-bearing species, including 7 cultivated species. Spooner and Salas [2] reduced the number further to 189 species (including only 1 cultivated species). Phylogenetic and phenetic analysis of previous studies, reviewed in van den Berg and Jacobs [3] and Jacobs et al. [9] revealed that accessions from many wild *Solanum *species, especially the species of the South American series *Tuberosa*, *Megistacroloba*, and *Yungasensia*, are closely related. This is consistent with the observations that they freely exchange genes and produce hybrids under artificial conditions. Because of this, we chose as the starting point of our analysis the AFLP data used by Jacobs et al. [9] to consider the individual plants as belonging to one gene pool, rather than to separate taxa, and to employ a population genetics approach to detect the genetic structure of these AFLP data for the group of South American representatives of *Solanum *section *Petota.*

To test which accessions may belong to one or more species groups we used a Bayesian population clustering approach implemented in the program STRUCTURE 2.2 [10,11]. STRUCTURE clusters individuals without using a-priori information about their identity. The primary assumptions of the model used in STRUCTURE are Hardy-Weinberg equilibrium (HWE) within populations and linkage equilibrium among loci, and the program attempts to find population groupings that are not in disequilibrium [11]. Both assumptions may not always be valid when taking a more or less random set of accessions collected over a larger area as representing a species, but disequilibrium will always be smaller within a species than between species. The program has been successfully used in a large variety of population genetic studies, for example in the research of genetic structure in the human population [12], in the phylogeography of the sand-dune shrub *America pungens *[13], for distinguishing chicken breeds [14], and to detect hybrids between cultivated and wild apple [15,16]. Recently, STRUCTURE was also used in studies on phylogenetic relationships among birch species [17], on species delimitation in a recent species radiation in turtles [18] and in the Mexican jay [19], and produced part of the evidence for a separate species status of the Galapagos sea lion [20].

Accessions within one species are expected to share more alleles with each other than with accessions from other species. As a result, genetic differentiation among species is expected to be higher than within species. Consequently, if we subdivide an unstructured set of accessions according to their species labels, the fraction of the genetic variation present among, rather than within, groups will be higher if those species labels correctly identify the accessions. If the species labels are incorrect then combining accessions with incorrect species labels into new groups will increase the fraction genetic variation among the groups.

Thus, the genetic differentiation among alternative groupings as expressed in Fst values will allow us to further subdivide the groups resulting from the STRUCTURE analysis, and to distinguish genetically separate species from species that should be grouped together. This approach of species delimitation resembles somewhat the view of Shaffer and Thompson [18] that follows Mayden [21] and de Queiroz [22,23], in that they consider species as segments of evolutionary lineages. In this view, species delimitation comes down to identification of metapopulation-like lineages. The metapopulation lineage species definition leads to operational species delimitation approaches that recognize sets of populations that freely exchange genes in nature but have no or very restricted gene exchange with other sets of populations [18]. In this paper we describe how this approach works out for *Solanum *section *Petota.*

## Methods

### Plant Material

We used the plant material from the genus *Solanum *section *Petota *as described in Jacobs et al. [9], which consists of 4929 genotypes representing 916 accessions. From each accession a representative genotype was chosen [9]. A subset (out of the 916) consisting of 566 plants (one plant per accession) was made, representing the 89 species/subspecies from South America that appeared in the large polytomy of the trees presented by Jacobs et al. [9], plus the accessions that do not belong to the species groups with high jackknife or bootstrap support (viz. excluding the following supported groups: Acaulia group, Mexican diploid group, diploid Piurana group, tetraploid Piurana group, polyploid Conicibaccata group, diploid Conicibaccata group, Circaeifolia group, Longipedicellata group, and Iopetala group). Information on the accession numbers and geographic origin of these 566 samples can be found in Additional file 1. The nomenclature of the plant material follows that of Jacobs et al. [9]. This means that in some cases we have retained the original labels, even when taxonomic references suggested a change of the species name. However, a number of obvious mistakes (due to mislabeling) that became clear after preliminary AFLP analyses have been corrected after morphological examination.

### AFLP

The protocol of Vos et al. [24] was used to generate AFLP fragments. The plant material was fingerprinted with two *Eco*RI/*Mse*I AFLP primer combinations: E32/M49 and E35/M48. These primer combinations gave 91 and 131 polymorphic bands, respectively. The AFLP analysis was done on a MegaBACE 2.1 by Keygene N.V. Bands were scored as dominant markers, using the Keygene proprietary software.

### Data analysis

#### Bayesian clustering

The 566 South-American accessions were analyzed with STRUCTURE 2.2 [10,11] in an 'unsupervised' procedure according to Rosenberg [25] based on genetic similarities only. We used the approach of coding the dominant markers as described by Falush et al [26]. The dominant AFLP data were entered by coding both alleles as '1' when the AFLP band was present and both as '0' when the band was absent. We specified '0' as the recessive allele for all the AFLP data. This enables the simultaneous analysis of accessions with different levels of ploidy like described by Schenk et al. [17]. Evanno et al. [27] showed that results of AFLPs with STRUCTURE can be as accurate as those of microsatellites. Estimates for the log likelihood were obtained using the admixture model and the assumption that the allele frequencies are correlated. The log likelihood estimates were obtained for 10 replicate runs at each K ranging from K = 1 to K = 30. For each run, we used a burn-in of 25,000 cycles and a data run of 100,000 cycles.

To test whether STRUCTURE was suitable for analyzing the *Solanum *AFLP data, a pilot analysis was carried out on the condensed dataset of 916 individuals. Almost all species groups as defined by Jacobs et al. [9] and smaller supported branches in the NJ tree have their own cluster at K = 18 or higher (results not shown), which confirms that STRUCTURE can be used for the AFLP dataset.

#### Partitioning of genetic variation within and among groups

It is unrealistic to assume that one STRUCTURE analysis could separate all species. Some of the 566 accessions may be from a genetically homogeneous species that occupies a small area, while others may be from a genetically highly variable species that occupies a large area. Some species were represented by many accessions, others by only a few. Therefore, while increasing the number of clusters (K) in the STRUCTURE analyses, accessions of certain species may already start to be assigned to different clusters before accessions of other species would be separated from each other. When large datasets are analyzed convergence problems for the Gibbs sampler algorithm used in STRUCTURE software may occur [12,28]. Therefore we decided do a nested analysis.

The second level (nested) analyses could be done again by STRUCTURE for each group separately, as e.g. Jing et al. [29] did in *Pisum*. The advantage is that an a priori grouping is made and accessions formerly classified under the same name may end up in different groups. An alternative option was to optimize the grouping of accessions by maximizing the Fst among the species or among combinations of species. This has two important advantages: (1) all plants within an accession can be included in this computationally simple analysis, and (2) even if several rounds of grouping are performed, it is still much faster than optimizing and performing a STRUCTURE analysis on each of the 16 clusters. A disadvantage is that accessions of the same name remain together, which may mean that in theory the best solution is less optimal than obtained with the nested STRUCTURE approach.

As a pilot experiment, we performed a nested STRUCTURE analysis on a few clusters and compared the results to an Fst analysis of the same clusters. The results were compared by calculating the Fst among groups for the nested STRUCTURE analysis and for the optimized Fst approach. The optimized Fst approach always resulted in a higher value for the Fst among the groups within the cluster (not shown). We therefore decided to continue with the Fst analysis. This combination is a novel approach.

The partitioning of genetic variation (Fst) among STRUCTURE clusters or among new groups within a cluster was computed using AFLP-SURV 1.0 [30]. The allelic frequencies at AFLP loci were calculated from the observed frequencies of fragments, using the Bayesian approach [31] (assuming diploid species and Hardy-Weinberg equilibrium) using all 2767 available genotypes for the 566 accessions (when available 5 plants per accession). We assumed a uniform prior distribution of allelic frequencies. Significance of the Fst values was tested by 1000 permutations. The confidence limits obtained were used to determine the significance of differences between the separate estimates.

#### Grouping within clusters by maximizing Fst

Within each of the 16 STRUCTURE clusters we calculated Fst based on the species present using AFLP-Surv. Subsequently, combinations of accessions with different species labels were made and the overall Fst value and pairwise Fst values between the groups within a cluster were computed. We performed several rounds of grouping. Each time the accessions of those species or groups that showed a pairwise Fst of less than the observed overall Fst of the groups within the cluster were combined. This process was repeated, merging species and species groups, until further merging of groups did not increase the overall Fst value significantly.

## Results

### Clustering of the 566 South-American accessions into 16 clusters

**Figure 1:**
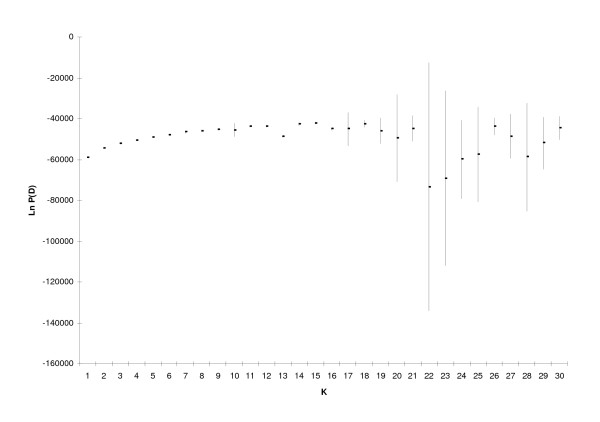
**Mean Ln P(D) ±SD for 10 replicate runs at each level of K proposed clusters**.

The 566 South-American accessions were analyzed using STRUCTURE, testing various numbers of groups, from K = 1 to K = 30. Figure 1 shows the average posterior probability Ln(P(D)) for 10 runs as a function of K. The posterior probability increases until around K = 16, after which it reaches a plateau. From K = 18 onwards the posterior probability became increasingly variable among runs, and the clustering of accessions became unstable between replicate runs. In contrast, at K = 16 the clustering results were stable and most clusters had the same composition in all 10 replicate runs. We therefore took K = 16 (Ln P(D) = -41181.7) as the optimal K.

**Figure 2:**
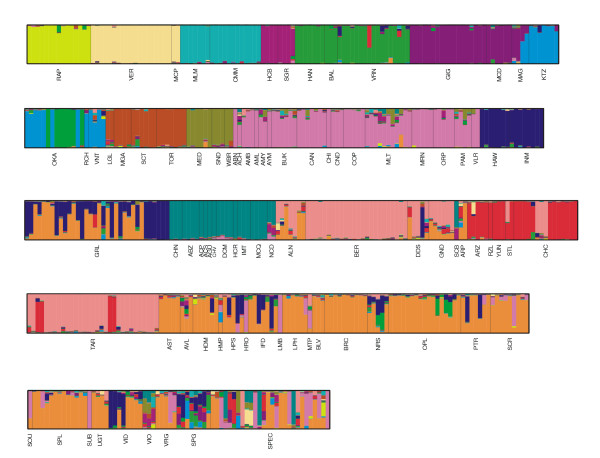
**Estimated population structure for K = 16**. Each accession is represented by a thin line, which is partitioned in K colored segments that represent the membership to K clusters. The labels below indicate the species labels.

The estimated population structure of one run at K = 16 is shown in Figure 2. Each individual accession is presented by a thin vertical line, and this line shows colored segments that represent the relative percentage of membership to the K clusters (the underlying data can be found in Additional file 1). The accessions labeled as *S. okadae*, *S. raphanifolium*, *S. verrucosum*, and *S. macropilosum *occupy exclusively one cluster, while many other accessions are found to share a cluster with accessions from one or more other species, for instance *S. huancabambense *with *S. sogarandinum*. Many accessions labeled with the same species name are distributed over two clusters, e.g. the accessions of *S. maglia, S. gourlayi, S. tarijense*. Finally, there is a number of species whose accessions show membership to more than two clusters. Additional file 1 provides the detailed results on the composition of the clusters and the percentage of membership per individual accession for these clusters, in the run with the highest probability. Most clusters defined by STRUCTURE for K = 16 are the same in all 10 runs. The main exception is cluster 3, which was found in only 3 out of 10 runs as a separate unit. In the other 7 runs its accessions were combined with those of cluster 4.

The partitioning of genetic variation among the clusters (Fst) in the 16 cluster arrangement represented 31% of the genetic variation (Table 1). For comparison, we also calculated that the 89 pre-existing taxa explained 29% of the existing genetic variation. A subdivision in 10 groups (one run of a suboptimal STRUCTURE analysis at K = 10) already explained 27%. The 566 individual accession arrangement showed the lowest value of Fst, as only 15% of the genetic variation is present among accessions. All Fst values were significantly different from each other.

**Table 1:** Genetic differentiation in complete dataset

	N^*^	Ht	Hw	Hb	Fst	p-value
among the accessions	538	0.3256	0.2783	0.0473	0.1453	<0.001
among the old species labels	89	0.2632	0.1855	0.0777	0.2953	<0.001
among the clusters at k = 16	16	0.2077	0.1430	0.0647	0.3124	<0.001
among the clusters at k = 10	10	0.2023	0.1475	0.0548	0.2733	<0.001
among new subgroups (see table 2)	44	0.2438	0.1594	0.0844	0.3464	<0.001

*) 28 accessions labeled unknown species were excluded in this analysis.

n = number of sampled accessions/groups; Ht = total diversity; Hw = within population diversity; Hb = between population diversity; Fst = Wright's fixation index, differentiation among populations.

The level of genetic differentiation among the accessions was lower within the clusters than among the clusters (Table 2). The lowest values are for cluster 1, 6 and 15, which mainly or exclusively consist of accessions with only one species label, e.g. cluster 15, which contains only *S. okadae *accessions, has an Fst of 0.0029.

**Table 2:** Genetic differentiation within the 16 clusters

Cluster	species included in cluster	sampling units	n	Ht	Hw	Hb	Fst	species included in the clusters/new arrangements
**1**	rap (15)	among accessions	15	0.2679	0.2776	-0.0098	-0.0365	

**2**	ver (19), mcp (2) spec 262, spec 287	among accessions	23	0.2515	0.2448	0.0068	0.0272	

		among species	4	0.2765	0.2063	0.0702	0.2377	

		among new groups	3	0.2445	0.1551	0.0894	0.3598	

		among new groups	***2***	***0.2859***	***0.1471***	***0.1388***	***0.4675***	***(ver, mcp) (spec 262, spec287)***

**3**	ktz(7), mag (2), oka (8), rch (1), spg (1), vnt (4)	among accessions	22	0.2875	0.2704	0.0171	0.0592	

		among species	6	0.2438	0.2113	0.0325	0.1285	

		among new groups	***3***	***0.2***	***0.1527***	***0.0473***	***0.2319***	***(ktz, mag, rch) (oka, vnt) spg***

		among new groups	2	0.1686	0.1305	0.0381	0.2231	

**4**	gig(18), mag (2), mcd (6) spg (1)	among accessions	27	0.2556	0.2457	0.0098	0.0384	

		among species	4	0.1973	0.1777	0.0195	0.0984	

		among new groups	***3***	***0.213***	***0.1898***	***0.0231***	***0.1059***	***(gig, mcd) mag spg***

		among new groups	2	0.3341	0.3064	0.0277	0.0639	

**5**	med (6), snd (3), wbr (2) vio (1)	among accessions	13	0.303	0.2864	0.0166	0.0548	

		among species	4	0.2594	0.2069	0.0525	0.2023	

		among new groups	3	0.2626	0.1949	0.0678	0.2559	

		among new groups	***2***	***0.2926***	***0.2055***	***0.0871***	***0.2893***	***(snd, wbr, med) vio***

**6**	cmm (12), mlm (7)	among accessions	19	0.3357	0.3535	-0.0177	-0.0528	

		among species	***2***	***0.1763***	***0.1395***	***0.0368***	***0.2084***	***cmm mlm***

**7**	abz (4), acp (1), acs (1), agu (1), chn (3), chv (1), dcm (4), hcr (1), imt (3), mcq(4), ncd (2), scb (1) vio (1) spec 205, spec 310, spec 6	among accessions	30	0.3016	0.2449	0.0567	0.1878	

		among species	16	0.2754	0.1988	0.0766	0.2777	

		among new groups	7	0.2448	0.143	0.1019	0.4175	

		among new groups	6	0.245	0.1405	0.1045	0.4276	

		among new groups	***5***	***0.2513***	***0.1419***	***0.1094***	***0.4357***	***(acs, agu, hcr, acp, scb, chn, mcq, imt, spec 205, spec 310, spec 6) (dcm, chv) ncd, abz, vio***

		among new groups	4	0.25	0.1418	0.1082	0.4333	

**8**	hcb(4), sgr (3), vio (1)	among accessions	9	0.2813	0.2319	0.0494	0.1758	

		among species	***3***	***0.2774***	***0.1919***	***0.0856***	***0.3043***	***hcb, sgr, vio***

		among new groups	2	0.3053	0.2128	0.0925	0.2948	

**9**	bal(3), han (7), vrn (16), spg (1)	among accessions	27	0.2659	0.2434	0.0226	0.0847	

		among species	4	0.2092	0.1687	0.0405	0.1904	

		among new groups	3	0.2133	0.1665	0.0468	0.2114	

		among new groups	***2***	***0.1662***	***0.1251***	***0.0411***	***0.2459***	***(bal, vrn, spg) han***

**10**	abn (1), ach(1), amb (3), aml(1), amy (2), aym (1), buk (6), can (7), chi (1), cnd (3), cop (5), hro (1), lmb (1), mlt (11), mrn (5), orp (5), pam (4), sou (1), scr (1), sub (1) vlr (2) vrg (1) spec 998, spec 184, spec 292, spec 533, spec 726, spec 796, spec 394, spec 933	among accessions	72	0.3095	0.2881	0.0213	0.069	

		among species	31	0.2828	0.2529	0.03	0.1058	

		among new groups	16	0.2598	0.2171	0.0427	0.1645	

		among new groups	7	0.2574	0.2045	0.0529	0.2066	

		among new groups	5	0.2796	0.2195	0.0601	0.2143	

		among new groups	***4***	***0.2996***	***0.2336***	***0.066***	***0.2194***	***(abn, ach, amb, aml, amy, aym, buk, can, chi, cnd, cop, hro, lmb, mlt, mrn, orp, pam, sou, scr, sub, vlr, vrg, spec 998, spec 184, mtp, spec 292, spec 533, spec 726, spec 796) ach, spec933, spec394***

		among new groups	3	0.2748	0.2142	0.0606	0.2189	

**11**	lgl (2), mga (4), sct (6), tor (7)	among accessions	19	0.3231	0.31	0.0131	0.0401	

		among species	4	0.2285	0.1885	0.04	0.1743	

		among new groups	***3***	***0.2421***	***0.1968***	***0.0453***	***0.1858***	***(mga, tor) lgl, sct***

		among new groups	2	0.2269	0.1877	0.0391	0.1704	

**12**	arz (5), chc (12), rzl (1), stl(1), yun (3), tar (4), vrn (1) spec 210, spec211, spec329, grl(1)	among accessions	31	0.2927	0.2716	0.0211	0.072	

		among species	11	0.2784	0.2506	0.0278	0.0991	

		among new groups	6	0.2236	0.1867	0.0369	0.1646	

		among new groups	4	0.22	0.178	0.042	0.1886	

		among new groups	***3***	***0.2317***	***0.1853***	***0.0465***	***0.1949***	***(chc, rzl, stl, grl, spec210, spec211, spec329, yun, arz) vrn, arz***

		among new groups	2	0.1772	0.1445	0.0326	0.1831	

**13**	dds (1), grl (15), hps (3), ifd (2), inm (8), ptr (3), vid (3), nrs (2), spg (3), haw (7)	among accessions	47	0.3045	0.2885	0.016	0.0526	

		among species	10	0.2252	0.18	0.0452	0.2006	

		among new groups	***5***	***0.2135***	***0.1582***	***0.0553***	***0.259***	***(grl, dds, hps, ifd, inm, ptr) vid, haw, spg, nrs***

		among new groups	4	0.2066	0.1545	0.0521	0.2522	

**14**	chc(3), dds((3), tar(27), aln(4), ber(23), gnd(5), stl(1), spec 255, spec 601	among accessions	69	0.2671	0.2518	0.0153	0.0573	

		among species	9	0.2092	0.1823	0.027	0.1276	

		among new groups	6	0.1726	0.148	0.0246	0.1417	

		among new groups	5	0.1766	0.1502	0.0264	0.1479	

		among new groups	***4***	***0.157***	***0.1327***	***0.0244***	***0.1549***	***(stl, spec 255, spec 601, yun, chc, dds, tar) aln ber gnd***

		among new groups	3	0.1616	0.1383	0.0233	0.1439	

**15**	oka (7)	among accessions	***7***	***0.261***	***0.2602***	***0.0007***	***0.0029***	

**16**	grl (18), hps(2), aln(3), arp(2), dds(1), hmp(1) ptr(5), scr(8), lph(6), ugt(4), vrg(4), opl(17), ifd(5), vid(5), gnd(1), vrn(1), nrs(2), spg(1), spl(13), brc(10), ast(5), blv(3), hdm(6), avl(3) spec352, spec43, spec123, spec165, spec891, spec381, spec649	among accessions	134	0.3169	0.302	0.015	0.0473	

		among species	31	0.2456	0.215	0.0306	0.1245	

		among new groups	14	0.217	0.1823	0.0347	0.1597	

		among new groups	7	0.214	0.1742	0.0399	0.1855	

		among new groups	6	0.2166	0.1749	0.0418	0.1913	

		among new groups	***5***	***0.224***	***0.1799***	***0.0442***	***0.1952***	***(grl, hps, aln, arp, dds, hmp ptr, scr, lph, ugt, vrg, opl, ifd, vid, gnd, vrn, nrs, spg, spl, brc, spec416, spec352, spec43, spec123, spec165, spec891, spec381), (ast, blv), hdm, avl, spec649***

		among new groups	4	0.2372	0.1932	0.0439	0.1831	

n = number of sampled accessions; Ht = total diversity; Hw = within population diversity; Hb = between population diversity; Fst = Wright's fixation index, differentiation among populations. The explanation of the species code can be found in table 3.

Genetic differentiation among species within clusters that contain accessions from two species ranged from 9.8% in cluster 4 to 27.8% in cluster 7. In cluster 4, cluster 10, and cluster 12 the species arrangement only added a small part to the genetic differentiation, relative to the value for all accessions separately.

### Further subdivision of the 16 clusters

As the contribution to the partitioning of genetic variation could differ for the various species within a cluster, we performed several rounds of grouping on all 2767 individuals available for these accessions. Each time the accessions of those species that showed a pairwise Fst of less than the observed overall Fst of the groups within the cluster were combined into one group, so that in the next round the number of groups was lower. The process was repeated, merging species and species groups, until further merging did not increase the Fst value. Table 2 lists the Fst value of the optimal number of groups, along with those of the value obtained with one group more or less, and the group structure of the optimal configuration is reported. In most of the clusters one or two merging steps were sufficient to reach a maximum Fst, but in cluster 7, 12, and 14, three cycles were needed, while in cluster 10 and 16 the process took four cycles. In some clusters the highest overall Fst was reached when most of the species labels were merged together; this was the case in cluster 10, 14 and 16. In other clusters the optimal Fst was reached at an arrangement that only merged a few of the species in the cluster, while other species remained separate. This was the case in cluster 3, 4 and 13. In cluster 8 no new arrangement yielded a higher Fst. Overall, the 566 accessions were grouped into 44 genetically distinct groups.

The assignment of the 566 accessions into 44 genetically distinct groups was then used to infer the support for the 89 species into which these accessions had been classified. The results are presented according to taxonomical classification in Table 3, and will be discussed below. For those species (18) that were represented by only one accessions in this study, no conclusion could be drawn. For 43 species there was no evidence, for 20 there was weak evidence and for 8 there was good evidence.

**Table 3:** Information on species labels and accessions used in the analysis and suggestions for species status

series according to Hawkes (1990)	species	species abbreviation	accessions code	source codes (genebank)	(total nr. of accessions in 566 dataset)	taxonomic remarks	evidence for species status according to the authors
*Tuberosa II*	*S. abancayense *Ochoa	abn	423	CGN 18357	1	synonym of *S. bukasovii *(Ochoa, 1999)	not enough accessions

*Piurana*	*S. albornozii *Correll	abz	2, 102, 103, 466	PI 561637, GLKS 35297, GLKS 35298, CGN 22731	4		weak evidence

*Tuberosa III*	*S. achacachense *Cárdenas	ach	99	GLKS 32830	1		not enough accessions

*Tuberosa II*	*S. ancophilum *(Correll) Ochoa	acp	304	CIP 761448	1	synonym of *S. rhomboideilanceolatum *Ochoa (Hawkes, 1990)	not enough accessions

*Tuberosa II*	*S. acroscopicum *Ochoa	acs	100	GLKS 32436	1		not enough accessions

*Tuberosa II*	*S. augustii *Ochoa	agu	305	CIP 762631	1		not enough accessions

*Tuberosa III*	*S. alandiae *Cárdenas	aln	257, 320, 455, 457, 458, 459, 460	CPC 7212, CGN 18245, CGN 22349, cgn962384, CGN 20651, CGN 18260, CGN 18264	7		weak evidence for combination with gnd

*Tuberosa II*	*S. ambosinum *Ochoa	amb	104, 105, 467,	GLKS 32282, GLKS 35299, CGN 18358	3		no evidence

*Tuberosa II*	*S. amabile *Vargas	aml	3	PI 365356	1	synonym of *S. canasense *(Hawkes, 1990)	not enough accessions

*Tuberosa II*	*S. amayanum *Ochoa	amy	302, 303	CIP 763004, CIP 763005	2		no evidence

*Megistacroloba*	*S. aracc-papa *Juz.	arp	109, 110	GLKS 30082, GLKS 30081	2	nomen dubium (Hawkes, 1990)	no evidence

*Yungasensa*	*S. arnezii *Cárdenas	arz	4, 111, 112, 113, 471	PI 545880, GLKS 32832, GLKS 32833, GLKS 32834, GLKS 32831	5		no evidence

*Megistacroloba*	*S. astleyi *Hawkes and Hjert.	ast	114, 472, 474, 475, 476	GLKS 32836, CGN 18207, CGN 18210, CGN 18211, CGN 18212	5		weak evidence for combination with blv

*Tuberosa III*	*S. avilesii *Hawkes and Hjert.	avl	477, 478, 479,	CGN 18255, CGN 18256, CGN 18257	3		no evidence

*Tuberosa II*	*S. aymaraesense *Ochoa	aym	5	PI 607896	1		not enough accessions

*Tuberosa III*	*S. vernei *subsp. *ballsii *(Hawkes) Hawkes and Hjert.	bal	906, 907, 908	CGN 17992, CGN 17993, CGN 17994	3		weak evidence for combination with vrn

*Tuberosa III*	*S. berthaultii *Hawkes	ber	322, 323, 324, 480, 481, 482, 483, 484, 485, 486, 487, 488, 489, 490, 491, 492, 493, 494, 561*, 939, 940, 941, 943, 944	CGN 20644, CGN 20650, CGN 18042, CGN 18074, CGN 18190, CGN 20635, CGN 20636, CGN 22715, CGN18216, CGN 22716, CGN 20645, CGN 18246, CGN 23804, CGN 18228, CGN 22727, BGRC 15479, CGN 17823, CGN 18118, GLKS 31670*, CGN 18189, CGN 23508, CGN 18267, CGN 17716, CGN 23477	24		no evidence

*Megistacroloba*	*S. boliviense *Dunal	blv	496, 498, 499	CGN 18196, CGN 18070, INTA 73228B	3		weak evidence for combination with ast

*Tuberosa III*	*S. brevicaule *Bitter	brc	327, 505, 506, 507, 509, 1020, 1025, 1026, 1040, 1047,	CGN 18231, CGN 17841, CGN 18226, CGN 18232, CGN 22321, CGN 18030, CGN 18223, CGN18247, CGN22322, CGN22717	10		no evidence

*Tuberosa II*	*S. bukasovii *Juz.	buk	328, 511, 512, 514, 955, 971,	CGN 17683, CGN 17684, CGN 17737, CGN 17821, CGN 21305, CGN 17738	6		no evidence

*Tuberosa II*	*S. canasense *Hawkes	can	260, 526, 527, 528, 529, 951$, 952, 953	CPC 2725, cgn960639, CGN 17722, CGN 17672, CGN 17589, CGN 20592$, CGN 18072, CGN23007	7		no evidence

*Yungasensa*	*S. chacoense *Bitter	chc	125, 126, 127, 246*, 263, 338, 470$, 543, 544, 545, 546, 547, 548, 549, 550, 551	GLKS 30162, GLKS 30161, GLKS 30180, GLKS 32343*, CPC5901, CGN 18248, CGN 17679$, cgn962709, CGN 18365, CGN 17702, CGN 22384, CGN 18202, CGN 18294, CGN 18338, cgn961764, CGN 22368	15		no evidence

*no information*	*S. chillonanum *Ochoa	chi	12	PI 607890	1		not enough accessions

*Tuberosa II*	*S. chancayense *Ochoa	chn	1, 552, 553	VIR 20892, CGN 18036, CGN 18356	3		no evidence

*Megistacroloba*	*S. chavinense *Correll	chv	11	PI 498235	1		not enough accessions

*Commersoniana*	*S. commersonii *Dunal	cmm	265, 575, 576, 577, 578, 1017, 1018, 1019, 1027, 1028, 1039, 1050	CPC 5861, cgn961592, cgn961597, CGN 18027, CGN 22351, CGN 17988, CGN 18024, CGN 18026, CGN 18327, CGN 18328, GLKS 35340, CGN 23492	12		evidence

*Tuberosa III*	*S. candolleanum *P. Berthault	cnd	530, 531, 532	PI 498226, CGN 18132, CGN 20603	3		no evidence

*Tuberosa II*	*S. coelestipetalum *Vargas	cop	134, 135, 306, 307, 572	GLKS 35433, GLKS 35434, CIP 761755, CIP 761999, CGN 20557	5		no evidence

*Tuberosa II*	*S. dolichocremastrum *Bitter	dcm	147, 148, 149, 308,	GLKS 32342, GLKS 35348, GLKS 35349, CIP 762533	4		weak evidence for combination with chv

*Tuberosa III*	*S. x doddsii *Correll	dds	144, 145, 146, 588, 589,	GLKS 32882, GLKS 32883, GLKS 32880, CGN 20661, CGN 18359	5		no evidence

*Tuberosa III*	*S. microdontum subsp. gigantophyllum *(Bitter) Hawkes and hjert.	gig	361, 362, 710, 711, 712, 713, 714, 715, 956, 957, 960, 961, 962, 963, 964, 965, 966, 967,	CGN 18046, CGN 18083, CGN 18199, CGN 20639, CGN 18200, CGN 17595, CGN 23050, CGN 21342, CGN 18295, CGN 23511, CGN 20586, CGN 18048, CGN 17597, CGN 18049, CGN 18084, CGN 18003, CGN 18067, CGN 22372	18	synonym of *S. microdontum *Bitter (van den Berg and Spooner, 1992)	no evidence, part of microdontum

*Tuberosa III*	*S. gandarillasii *Cárdenas	gnd	16, 62, 163, 270, 346, 603,	PI 597750, PI 597751, GLKS 32423, CPC 7044, CGN 20560, CGN 17590	6		weak evidence for combination with aln

*Tuberosa III*	*S. gourlayi *Hawkes	grl	347, 604, 605, 606, 607$, 608, 609, 610, 611, 1000$, 1005, 1006, 1008, 1009, 1010, 1011, 1012, 1013, 1014, 1015, 1021, 1022, 1029, 1030, 1032, 1033, 1034, 1035, 1036$, 1037, 1042, 1043$, 1044, 1048, 1049, 1051, 1052$, 1053, 1054, 1055$	CGN 17851, CGN 22705, CGN 17591, CGN 18039, CGN 22380$, cgn961345, CGN 17592, CGN 22336, CGN 21335, cgn961607$, CGN 17872, CGN 17873, CGN 17962, CGN 17963, CGN 17965, CGN 17966, CGN 17967, CGN 17969, CGN 17970, CGN 17971, CGN 18065, CGN 18066, CGN 20585, CGN 20594, CGN 20657, CGN 21332, CGN 21333, CGN 21334, CGN 21336$, CGN 21341, CGN 22340, CGN 22342$, CGN 22343, CGN 23022, CGN 23486, CGN 23497, CGN 23515$, cgn960071, cgn961347, CGN 23794$	34	synonym of *S. leptophyes *(Ochoa, 1990)	no evidence

	*S. hannemanii*	han	252*, 628, 629, 630, 631, 632, 633	GLKS 32196*, CGN 17996, CGN 17854, CGN 17997, CGN 20578, CGN 17856, CGN 17858	7	provisional name	weak evidence

	*S. hawkesianum*	haw	166, 167, 634, 635, 636, 637, 638,	GLKS 32762, GLKS 32765, CGN 17888, CGN 17889, CGN 17890, CGN 17891, CGN 17892	7	provisional name	weak evidence

*Yungasensa*	*S. huancabambense *Ochoa	hcb	18, 170, 353, 354	PI 365359, GLKS 32441, CGN 18306, CGN 17719	4		evidence

*Piurana*	*S. hypacrarthrum *Bitter	hcr	311	CIP 761259	1		not enough accessions

*Tuberosa III*	*S. hondelmannii *Hawkes and Hjert.	hdm	168, 351, 644, 645, 646, 647, 648	GLKS 32852, CGN 18106, cgn961918, cgn962199, CGN 18192, CGN 18193, cgn962204	7		weak evidence

*Tuberosa II*	*S. humectophilum *Ochoa	hmp	171	GLKS 32829	1		not enough accessions

*Tuberosa III*	*S. hoopesii *Hawkes and Okada	hps	169, 650, 651, 652, 653	GLKS 32885, CGN 18363, CGN 18367, CGN 18368, CGN 18372	5		no evidence

*Tuberosa II*	*S. huarochiriense *Ochoa	hro	309	CIP 761224	1		not enough accessions

*Megistacroloba*	*S. infundibuliforme *Phil.	ifd	664, 665, 666, 667, 668, 1007, 1023	CGN 17720, CGN 23063, CGN 22334, CGN 23048, cgn960696, CGN 17959, CGN 18079	7		no evidence

*Tuberosa II*	*S. immite *Dunal	imt	63, 64, 172,	PI 498245, PI 365331, GLKS32819	3		no evidence

*Tuberosa III*	*S. incamayoense *K.A. Okada and A.M. Clausen	inm	657, 658, 659, 660, 661, 662, 663, 1016	CGN 18077, CGN 21320, CGN 17874, CGN 17875, CGN 17968, cgn961363, CGN 22335, CGN 17972	8		no evidence

*Tuberosa III*	*S. kurtzianum *Bitter and Wittm.	ktz	275, 276, 675, 676, 677, 678, 995,	CPC 5864, CPC 5889, CGN 22338, cgn961563, CGN 23042, cgn961013, CGN 22353	7		weak evidence

*Lignicaulia*	*S. lignicaule *Vargas	lgl	179, 685	GLKS 32215, CGN 17723	2		weak evidence

*Conicibaccata*	*S. limbaniense *Ochoa	lmb	686	CGN 22720	1		not enough accessions

*Tuberosa II*	*S. leptophyes *Bitter	lph	356, 357, 680, 682, 683, 684,	CGN 18174, CGN 18140, CGN 18173, CGN 18167, CGN 20611, CGN 18126	6		no evidence

*Maglia*	*S. maglia *Schtdl.	mag	75, 76, 359, 688,	PI 245087, PI 558316, CGN 18064, CGN 22719	4		no evidence

*Tuberosa III*	*S. microdontum *Bitter	mcd	360, 707, 708, 958, 959, 994	CGN 17596, CGN 22382, CGN 18259, CGN 20646, CGN 18047, CGN 20597	6		evidence

*Tuberosa I*	*S. macropilosum *Correll	mcp	23, 74	PI 607844, PI 607845	2	synonym of *S. verrucosum *(Spooner et al. 2004)	no evidence, part of ver

*Tuberosa II*	*S. mochiquense *Ochoa	mcq	186$, 716, 717, 718, 719	GLKS 32319$, CGN 20587, CGN 18263, CGN 17731, CGN 21360	4		no evidence

*Tuberosa II*	*S. medians *Bitter	med	183, 691, 692, 693, 694, 695,	GLKS 32226, CGN 21349, CGN 18043, CGN18308, CGN 21343, CGN 18307	6		weak evidence

*Megistacroloba*	*S. megistacrolobum *Bitter	mga	696, 697, 699, 700	CGN 23064, CGN 17828, CGN 22347, CGN 20601	4		weak evidence for combination with tor

*Commersoniana*	*S. commersonii *subsp. *malmeanum *(Bitter)	mlm	139, 266, 579$, 580, 581, 1038, 1045, 1058	GLKS 35340, CPC 7520, CGN 18329$, CGN 18025, CGN 18215, CGN21353, CGN 22352, cgn962274	7		evidence (subspecies or species)

*Tuberosa II*	*S. multidissectum *Hawkes	mlt	363, 722, 723, 724, 725, 727, 728, 729, 730, 731, 732	CGN 17824, CGN 21344, CGN 18330, cgn960739, CGN 17686, CGN 17733, cgn960736, CGN 17825, cgn961613, cgn17840, cgn960967	11	synonym of *S. bukasovii *Juz. f. multidissectum (Hawkes) Ochoa	no evidence

*Tuberosa II*	*S. marinasense *Vargas	mrn	77, 181, 182, 277, 690	PI 607884, GLKS 35430, GLKS 32281, CPC 7172, CGN 17594	5		no evidence

*Tuberosa II*	*S. multiinterruptum *Bitter	mtp	190	GLKS 32431	1		not enough accessions

*Tuberosa III*	*S. neocardenasii *Hawkes and Hjert.	ncd	193, 734	GLKS 32855, CGN 18217	2		no evidence

*Tuberosa III*	*S. neorossii *Hawkes and Hjert.	nrs	281, 735, 736, 737, 987*	CPC 6047, CGN 18280, CGN 17599, CGN 18051, CGN 17763*	5		no evidence

*Tuberosa III*	*S. okadae *Hawkes and Hjert.	oka	283, 365*, 366, 367, 368, 739, 740, 741$, 742, 743, 744, 745, 746, 969, 970	CPC 7129, CGN 18000*, CGN 18109, CGN 18108, CGN 17998, CGN 18269, CGN 17999, CGN 18279$, cgn962076, cgn962078, CGN 18157, CGN 22709, CGN 18129, CGN 22703, CGN 20599	14		evidence

*Tuberosa III*	*S. oplocense *Hawkes	opl	747, 749, 750, 751, 752, 753, 754, 1001$, 1002, 1003, 1004, 1024, 1031, 1041, 1046, 1056, 1057, 1059	CGN 23049, cgn962217, CGN 21352, CGN 18088, CGN 18085, CGN 21319, CGN 17736$, CGN 17868, CGN 17869, CGN 17870, CGN 18087, CGN 20638, CGN 22324, CGN 22713, CGN 23798, cgn961876, cgn962541	17		no evidence

*Tuberosa II*	*S. orophilum *Correll	orp	29, 83, 84, 196, 756	PI 498213, PI 498209, PI 498212, GLKS 35301, cgn962570	5		no evidence

*Tuberosa II*	*S. pampasense *Hawkes	pam	288, 762, 763, 764	CPC 6024, CGN 962604, CGN 20575, cgn960051	4		no evidence

*Tuberosa III*	*S. gourlayi subsp. pachytrichum × S. leptophyes*	ptr	612, 613, 614, 615, 616, 617, 618,	cgn18102, cgn18176, bgrc27294, bgrc27295, cgn18188, bgrc7231, bgrc28084	7	synonym of *S. leptophyes (*Ochoa, 1990)	no evidence

*Megistacroloba*	*S. raphanifolium *Cárdenas and Hawkes	rap	208, 209, 291, 380, 790, 791, 792, 793, 794, 797, 798, 799, 800, 801, 976	GLKS 30637, GLKS 30644, CPC 7090, CGN 17598, cgn960772, CGN 20589, CGN 18300, CGN 18089, cgn961878, CGN 18320, CGN 17752, CGN 18033, CGN 17833, CGN 17835, CGN 17822	15		evidence

*Tuberosa III*	*S. xrechei *Hawkes and Hjert.	rch	35	PI 558227	1		not enough accessions

*Tuberosa III*	*S. xruiz-lealii *Brücher	rzl	802	CGN 18117	1		not enough accessions

*Tuberosa II*	*S. scabrifolium *Ochoa	scb	37	PI 365363	1		not enough accessions

*Tuberosa III*	*S. xsucrense *Hawkes	scr	391, 843, 844, 845, 846, 847, 848, 849, 850	CGN 18205, CGN 20628, CGN 20630, CGN 20631, CGN 18187, CGN 20634, CGN 22350, CGN 18206, CGN 18105	9		no evidence

*Megistacroloba*	*S. sanctae-rosae *Hawkes	sct	803, 804, 805, 806, 807, 1061	CGN 20576, CGN 22344, CGN 17910, CGN 20564, CGN 17837, cgn961619	6		weak evidence

*Megistacroloba*	*S. sogarandinum *Ochoa	sgr	215, 315, 316, 814	GLKS 35382, CIP 761465, CIP 761586, CGN 17601	4		evidence

*Tuberosa II*	*S. sandemanii *Hawkes	snd	93, 94, 808	PI 607894, PI 607895, CGN 17600	3		weak evidence for combination with wbr

*Tuberosa II*	*S. soukupii *Hawkes	sou	815	CGN 18061	1	synonym of *S. canasense (*Hawkes 1990)	not enough accessions

*Tuberosa III*	*S. spegazzinii *Bitter	spg	217, 385, 386, 822, 823, 824, 826, 827, 828$	GLKS 32755, CGN 17759, CGN 17839, cgn960795, CGN 21318, CGN 22707, CGN 21321, CGN 23015, CGN 18034$	8		no evidence

*Tuberosa II*	*S. sparsipilum *(Bitter) Juz. and Bukasov	spl	382, 383, 384, 816, 817, 818, 819, 820, 821, 972, 973, 975, 978,	CGN 18225, CGN 18230, CGN 18154, CGN 18096, CGN 17838, CGN 18221, CGN 20653, CGN 17758, CGN 20602, CGN 18099, CGN 22702, CGN 18094, CGN 18131	13		no evidence

*Tuberosa III*	*S. xsetulosistylum *Bitter	stl	214, 811	GLKS 31014, CGN 20655	2		no evidence

*Tuberosa III*	*S. tarijense *Hawkes	tar	224, 225,280*,392, 852, 853, 854, 855, 856, 857, 858, 859, 860, 862, 863, 864, 865, 866, 867, 868, 869, 870, 871, 872, 873, 874, 875, 876, 877, 878, 879,	GLKS 31570, GLKS 31572, CPC 7208*, CGN 17861, CGN 22729, cgn962224, CGN 22714, CGN 18198, cgn960807, cgn960805, cgn960806, CGN 17975, cgn961432, CGN 21337, CGN 23795, cgn961736, CGN 17976, CGN 17974, CGN 17977, CGN 18107, cgn961128, CGN 17978, CGN 17979, cgn961441, CGN 17980, CGN 21338, CGN 17981, cgn961449, CGN 17982, cgn961451, cgn962690	31		no evidence

*Megistacroloba*	*S. megistacrolobum *subsp. *toralapanum *(Cárdenas and Hawkes)	tor	278, 701, 702, 703, 704, 705, 706	CPC 1773, CGN 17728, CGN 23006, CGN 18145, CGN 18146, CGN 18147, CGN 18125	7		weak evidence for combination with mga

*Tuberosa III*	*S. ugentii *Hawkes and K. A. Okada	ugt	44, 248, 249, 892,	PI 546029, GLKS 32887, GLKS 32889, CGN 18369	4		no evidence

*Tuberosa I*	*S. verrucosum *Schtdl.	ver	393, 825*, 909, 910, 911, 912, 914, 915, 916, 917, 918, 919, 920, 921, 922, 923, 988, 989, 990	CGN 17768, CGN 18100*, CGN 22326, CGN 22374, CGN 17764, CGN 20567, CGN 17769, CGN 17765, CGN 17773, CGN 17771, CGN 17766, CGN 17770, CGN 17772, cgn960832, cgn960833, CGN 20566, CGN 23017, CGN 17767, CGN 17774	19		evidence

*Tuberosa III*	*S. gourlayi *subsp. *vidaurrei (Cárdenas) Hawkes and hjert.*	vid	619, 620, 621, 622, 623, 624, 625, 626,	CGN 17848, CGN 17849, CGN 18040, CGN 17850, CGN 18038, CGN 17864, CGN 23024, CGN 23045	8		no evidence

*Conicibaccata*	*S. violaceimarmoratum *Bitter	vio	924, 925, 926,	CGN 18296, CGN 20647, CGN 22878	3		no evidence

*Tuberosa II*	*S. velardei *Ochoa	vlr	97, 893	PI 619114, CGN 18324	2		no evidence

*Tuberosa III*	*S. venturii *Hawkes and Hjert.	vnt	250, 894, 896, 993,	GLKS 32794, CGN 17761, cgn961508, CGN 17755	4		weak evidence

*Tuberosa III*	*S. virgultorum *(Bitter) Cárdenas and Hawkes	vrg	927, 928, 929, 930, 931, 932$	cgn962448, CGN 17775, cgn962072, CGN 20615, cgn962077, CGN 20652$	5		no evidence

*Tuberosa III*	*S. vernei *Bitter and Wittm.	vrn	895*, 897, 898, 899, 900, 901, 902, 903, 904, 905$, 979, 980, 981, 982, 983, 984, 985, 986	CGN 17762*, CGN 22728, CGN 18111, CGN 21350, CGN 22345, CGN 18112, CGN 18114, CGN 23039, CGN 18278, CGN 17836$, CGN 18110, CGN 21315, CGN 17995, CGN 18113, CGN 18115, CGN 23516, CGN 18277, cgn963094	17		weak evidence for combination with bal

*Tuberosa II*	*S. weberbaueri*	wbr	254, 300	GLKS 32725, CPC 6032	2		weak evidence for combination with snd

*Yungasensa*	*S. yungasense *Hawkes	yun	98, 934$, 935, 936	PI 614703, CGN 18336$, CGN 20677, CGN 20676	3		no evidence

## Discussion

Many described species in section *Petota *are very similar to each other and are able to cross, suggesting that this section is overclassified. We have tested this for the large group of South American species of the section *Petota*, using a population genetic approach that would allow us to identify any structure among this material, if present. The results obtained from the analysis of 566 South-American *Solanum *section *Petota *accessions with STRUCTURE showed an optimal overall subdivision of these accessions in 16 clusters. By maximizing the partitioning of genetic variation among groups (Fst) we obtained support for additional groups within these clusters, up to a total of 44 units (or 48 units including the unknown species accessions) (Table 2). This does not automatically mean that 44 is the correct number of species as genetic differentiation would be expected among separate species but it can also be found among populations within a species (see below). Nevertheless, the Fst values of the various species arrangements in Table 1 offer a clear indication of overclassification: Fst increases from 0.145 (the 566 accessions) to 0.273 (10 clusters) and to 0.312 (16 clusters). The highest value is obtained after the nested analysis, when 44 groups explain 35% of the genetic variation (the remainder being present within species). The Fst value of the 89 species arrangement (0.2953) is even lower than that of the 16 clusters (0.312), indicating that the current species arrangement is 'over the top' but still does explain a considerable part of the genetic variation within the dataset.

### Misclassification and overclassification

If not all accessions of a species are in one cluster but one or a few are present in different clusters, this may indicate misclassification. Occurrence of different species labels intermingled within one cluster points at overclassification. From both situations we see examples in our dataset and these may have consequences for the (sub) species status of the present taxa.

### Status of subspecies

In nearly all cases there was no support for maintaining taxa at the subspecies level. This is the case for the subspecies within the species *S. microdontum*, *S. vernei*, *S. boliviense *and *S. megistacrolobum*. Only one of the recognized subspecies was supported in our analysis: *S. commersonii *subsp. *malmeanum *could be differentiated genetically from *S. commersonii *subsp. *commersonii *(Table 3).

Some of these (sub) species have been extensively studied previously, using morphology. The subspecies *S. microdontum *subsp. *gigantophyllum *was already considered to be a synonym of *S. microdontum *[32] and should not be recognized, as this is a clear case of overclassification. Giannattasio and Spooner studied the boundaries between *S. megistacrolobum *subsp. *megistacrolobum *and *S. megistacrolobum *subsp. *toralapanum *using morphological data [33] and with molecular markers [34]. Based on their analysis they suggested to preserve *S. megistacrolobum *subsp. *toralapanum *as a distinct subspecies while our analysis does not find support for this. Spooner et al. [35] studied the relationships of *S. boliviense *and *S. astleyi *using RAPDs and concluded that *S. astleyi *should be reduced to a subspecies of *S. boliviense*. Our data do not provide support for a subspecies level in *S. boliviense.*

### Some species are supported

The following species are supported as genetically distinct units: *S. raphanifolium*, *S. verrucosum *(with *S. macropilosum *as synonym), *S. microdontum*, *S. commersonii*, *S. okadae *(only the seven accessions in cluster 15), *S. huancabambense*, and *S. sogarandinum*. The seven *S. okadae *accessions that appear in cluster 3 together with *S. venturii *accessions turned out to be mislabeled and have been corrected as being *S. venturii *accessions (personal communication R. Hoekstra, CGN). The accessions labeled *S. microdontum*, *S. huancabambense *and *S. sogarandinum *share their cluster with accessions from other species, but the optimal partitioning of genetic variation within the cluster shows that they represent distinct genetic units. This is consistent with the results from Jacobs et al. [9] and most of these species were also recognized in one or more other studies [2,6,32,36,37].

### Support for combinations of species, pointing at overclassification

Some species are assigned to one STRUCTURE cluster, but their accessions do not form distinct genetic units within the cluster on their own, but combined with accessions from another species they do (Table 2). These are probably cases of overclassification. Examples are the combination of *S. verrucosum *and *S. macropilosum *in cluster 2, of *S. kurtzianum *and *S. maglia *in cluster 3, of *S. venturii *and *S. okadae *in cluster 3, of *S. sandemanii*, *S. weberbauerii*, and *S. medians *in cluster 5. Some of these combinations have already been recognized in the literature, e.g. *S. macropilosum *is considered a synonym of *S. verrucosum *[6].

Spooner and Salas [2] recognized *S. medians *and *S. sandemanii*, but not *S. weberbauerii*, which name they apparently considered as a synonym (unfortunately, information about this was not provided). Spooner et al. [38] synonymized both *S. sandemanii *and *S. weberbaueri *under *S. medians*.

### Accessions scattered across clusters, pointing at mislabelling

The analysis showed that accessions from some species were scattered across two or even three clusters. This was the case for the accessions with the following species labels: *S. maglia*, *S. doddsii*, *S. chacoense*, *S. gourlayi*, *S. virgultorum*, *S. hoopesii*, *S. augustii*, *S. tarijense S. vernei*, *S. infundibuliforme*, *S. alandiae*, *S. neorosii*, *S. sucrense*, *S. pachytrichum*, and *S. violaceimarmoratum*. A major cause for this situation is probably mislabeling of accessions, although some of these species may be the product of hybridization events that occurred a long time ago. For instance, *Solanum doddsii *from Bolivia has been hypothesized to be a hybrid between *S. alandiae *and *S. chacoense *[39].

Misclassifications do occur since identification is often problematic due to ambiguous species characteristics. Problems with the identification of species were already addressed by Spooner and Salas [2] and Spooner and van den Berg [40], who noted that many of the taxa are extremely similar in morphology and many species are distinguished only by minor characters with often overlapping character states.

### Hybrid accessions

Many authors [1,2,4,41,42] have suggested that certain recognized species in *Solanum *sect. *Petota *are the results of hybridization. Recent hybridizations can readily be recognized from the STRUCTURE analysis by the probability with which they are assigned to a particular cluster. While most accessions have a very high probability (usually around 0.9) to belong to one cluster, hybrid individuals tend to have a much lower probability (< 0.5) and have a, often only slightly lower, probability to belong to another cluster. Schulte et al. [43] also argue that a posterior probability lower than 0.5 provides strong evidence for a recent hybrid origin of individuals.

To practically present our results, we have assigned all accessions to the cluster to which it had the highest probability, but Additional file 1 lists all probabilities for all accessions. Hybrid accessions thus identified include amongst others accessions of the species *S. spegazzinii *and *S. gourlayi*, which co-occur in northern Argentina. The *S. spegazzinii *accession SPG386 was assigned to cluster 3 with a probability of 0.459 and with 0.262 to cluster 16. Another example of recent hybridization is NRS737 which shows probabilities of 0.435 and 0.434 with the clusters 13 and 15, respectively. However, in all cases the actual parents are unknown.

### Non-supported species

Some species do appear in one cluster in the STRUCTURE analysis, but their accessions do not form a separate group in the Fst analysis, not even as part of a fixed combination with another species label. This concerns the following species: *S. mochiquense, S. immite, S. chancayense *in cluster 7, *S. canasense, S. bukasovii, S. candolleanum, S. coelestipetalum, S. pampasense, S. ambosinum, S. marinasense, S. multidissectum, S. velardei *in cluster 10, *S. arnezii, S. yungasense*, in cluster 12, *S. incamayoense *in cluster 13, *S. tarijense, S. berthaultii *in cluster 14, *S. arac-pappa, S. leptophyes, S. ugentii, S. oplocense, S. sparsipilum, and S. brevicaule *in cluster 16.

Many species mentioned in this category are part of what is termed the 'brevicaule complex' [7,8,44]: *S. canasense S. bukasovii, S. candolleanum, S. coelestipetalum, S. pampasense, S. ambosinum, S. marinasense, S. velardei, S. incamayoense, S. leptophyes, S. ugentii *and *S. sparsipilum*. Ugent [45] already proposed in 1970 that these should be reduced to one species. The division of the species according to our analysis in two clusters (10 and 16) reflects the presence of the northern and southern subgroups of the brevicaule-complex (see below). *Solanum oplocense *was shown to be a well-defined species using morphological data [7] and molecular data [8], but it was not distinct in an AFLP study [46] nor in ours. Previous results from a morphological study [47] and a more recent molecular study [48] had already suggested that the species *S. berthaultii *and *S. tarijense *should be combined. The species in cluster 7 were studied morphologically by Ames and collaborators [49], who placed *Solanum immite *and *S. chancayense *among the 6 distinctive species in a group of 29 species, the remainder of which were 'difficult to distinguish'.

### Clusters correspond to the geographical origin of the accessions

Many accessions within a cluster come from the same geographical region (Additional file 1). For the largest and most complicated clusters (7, 10, 12, 14, 16) the information on the geographic origin of the accessions allows to draw some tentative conclusions. Cluster 16 contains mostly accessions from Argentina and Bolivia from the southern brevicaule complex and cluster 10 consist mostly of accessions from Peru (and northern Bolivia) that can be considered as belonging to the northern brevicaule complex. This separation of the brevicaule complex in a northern and southern part was already noted by Kardolus [50], was confirmed by Spooner and Salas [2] and is accepted in the treatment of this group on the Solanaceae Source website (http://www.solanaceaesource.org), where Spooner and his collaborators maintain two species, *S. candolleanum *for the northern representatives, and *S. brevicaule *for the southern representatives. Cluster 7 contains almost exclusively Peruvian accessions, and some species labels in cluster 7 (*S. albornozii, S. augustii, S. chancayense, S. dolichocremastrum, S. immite*) are associated with series Piurana [1,2], but Jacobs et al. [9] could not find support for these species to be included in one of the recognized Piurana species groups. Ames and collaborators [49,51] studied putative members of series Piurana with, respectively, morphological data and COSII markers, and concluded that based on morphology only three out of a total of 33 species could be recognized. The molecular data supported more species, some of them lacking morphological support, and the authors announced that decisions on species boundaries will be formalized in a forthcoming taxonomic monograph.

Cluster 14 contains all *S. berthaultii *accessions and almost all *S. tarijense *accessions, plus a few accessions with other species labels, which mostly come from Bolivia and Argentina. Cluster 12 contains accessions from various geographical origins, most of them from Bolivia and Argentina but some are from Peru and Paraguay. This group may represent accessions that relatively easily exchanged genetic material. The geographical distribution of accessions within clusters is consistent with the notion that our approach produces a meaningful arrangement of the accessions into groups that may (have) exchange(d) genetic material. For exchange of genetic material at least the accessions with the different species labels should have overlapping or adjacent geographical areas, at present or in the recent past.

Indeed, information on the distribution areas of the species of sect. *Petota *given in Hijmans et al. [52] confirms overlapping areas for many species within the recognized clusters, e.g. the species *S. augustii, S. immite *and *S. dolichocremastrum *in cluster 7, and *S. berthaultii *and *S. tarijense *in cluster 14.

## Conclusion

A large number of species is presently recognized in the group of South American representatives of *Solanum *section *Petota*. The approach taken in the present paper was to determine the genetic distinctiveness of these species. The outcome questions the species and subspecies status of more than half of the taxonomic labels used in South American part of *Solanum *section *Petota*. The genetically distinct clusters and groups within clusters resulting from our analysis can be used as a basis for recognizing groups of species and for an evaluation of species status (Table 3).

## Authors' contributions

MJ carried out the analyses and drafted the manuscript, RVB, MS and BV participated in coordination and design of the study and in writing the manuscript. All authors read and approved of the final manuscript.

## Supplementary Material

Additional file 1 **Plant material used and cluster assignment**. This file contains information on the accession numbers and geographic origin of the 566 samples used in this study. Also indicated is the cluster to which an accession has been assigned. The table lists all probabilities for all accessions. In this file putative hybrid accessions may readily be detected through conditional formatting (probabilities above 0.5 are in dark grey cells, lower probabilities - that may be indicative of recent hybridisation - in white cells, and negligible probabilities in light grey font). Click here for file

## References

[B1] Hawkes JG The potato, Evolution, Biodiversity and Genetic Resources 1990 London. Belhaven Press

[B2] Spooner DM Salas A Gopal J, Khurana SMP Structure, Biosystematics, and genetic resources Handbook of potato production, improvement, and post-harvest management 2006 New York: The Haworth Press 1 39

[B3] van den Berg RG Jacobs MMJ Vreugdehil D Molecular Taxonomy Potato Biology and Biotechnology 2007 Oxford: Elsevier 55 76

[B4] Correll DS The potato and its wild relatives 1962 Renner: Texas Research Foundation

[B5] Spooner DM Hijmans RJ Potato systematics and germplasm collecting, 1989-2000 Am J Potato Res 2001 78 237 278 10.1007/BF02875691

[B6] Spooner DM van den Berg RG Rodríguez A Bamberg J Hijmans RJ Lara-Cabrera SI McPherson GD, Prather LA, Ranker TA, Reznicek AA Wild Potatoes (*Solanum *section *Petota*; *Solanaceae*) of North and Central America Systematic Botany Monographs 2004 USA: The American Society of Plant Taxonomists

[B7] van den Berg RG Miller JT Ugarte ML Kardolus JP Villand J Nienhuis J Spooner DM Collapse of morphological species in the wild potato Solanum brevicaule complex (*Solanaceae*: sect. *Petota*) Am J Bot 1998 85 92 109 10.2307/2446559 21684884

[B8] Miller JT Spooner DM Collapse of species boundaries in the wild potato *Solanum brevicaule *complex (*Solanaceae*, *Solanum *sect. *Petota*): molecular data Plant Syst Evol 1999 214 103 130 10.1007/BF00985734

[B9] Jacobs MMJ van den Berg RG Vleeshouwers VGAA Visser M Mank R Sengers M Hoekstra R Vosman B AFLP analysis reveals a lack of phylogenetic structure within *Solanum *section *Petota* BMC Evol Biol 2008 8 1 12 10.1186/1471-2148-8-145 18479504 PMC2413236

[B10] Falush D Stephens M Pritchard JK Inference of Population Structure Using Multilocus Genotype Data: Linked Loci and Correlated Allele Frequencies Genetics 2003 164 1567 1587 12930761 10.1093/genetics/164.4.1567 PMC1462648

[B11] Pritchard JK Stephens M Donnelly P Inference of Population Structure Using Multilocus Genotype Data Genetics 2000 155 945 959 10835412 10.1093/genetics/155.2.945 PMC1461096

[B12] Rosenberg NA Pritchard JK Weber JL Genetic structure of human populations Science 2002 298 2381 2385 10.1126/science.1078311 12493913

[B13] Pineiro R Aguilar JF Munt DD Feliner GN Ecology matters: Atlantic-Mediterranean disjunction in the sand-dune shrub *Armeria pungens *(*Plumbaginaceae*) Mol Ecol 2007 16 2155 2171 10.1111/j.1365-294X.2007.03280.x 17498238

[B14] Rosenberg NA Burke T Elo K Empirical Evaluation of Genetic Clustering Methods Using Multilocus Genotypes From 20 Chicken Breeds Genetics 2001 159 699 713 11606545 10.1093/genetics/159.2.699 PMC1461842

[B15] Coart E Vekemans X Smulders MJM Wagner I van Huylenbroeck J van Bockstaele E Roldán-Ruiz I Genetic variation in the endangered Wild apple (*Malus sylvestris *(L.) Mill.) in Belgium as revealed by AFLP and microsatellite markers. Consequences for conservation Mol Ecol 2003 12 845 857 10.1046/j.1365-294X.2003.01778.x 12753206

[B16] Koopman WJM Li Y Coart E van de Weg WE Vosman B Roldán-Ruiz I Smulders MJM Linked versus unlinked markers: multilocus microsatellite haplotype sharing as a tool to estimate gene flow and introgression Mol Ecol 2007 16 243 256 10.1111/j.1365-294X.2006.03137.x 17217342

[B17] Schenk MF Thienpont C Koopman WJM Gilissen LJWJ Smulders MJM Phylogenetic relationships in *Betula *(*Betulaceae*) based on AFLP markers Tree Genet Genom 2008 4 911 924 10.1007/s11295-008-0162-0

[B18] Shaffer HB Thomson RC Delimiting species in recent radiations Syst Biol 2007 56 896 906 10.1080/10635150701772563 18066926

[B19] McCormack JE Peterson AT Bonaccorso E Smith TB Speciation in the highlands of Mexico: genetic and phenotypic divergence in the Mexican jay (*Aphelocoma ultramarina*) Mol Ecol 2008 17 2505 2521 10.1111/j.1365-294X.2008.03776.x 18430143

[B20] Wolf JBW Tautz D Trillmich F Galápagos and Californian sea lions are separate species: Genetic analysis of the genus *Zalophus *and its implications for conservation management Front Zool 2007 4 20 10.1186/1742-9994-4-20 17868473 PMC2072946

[B21] Mayden RL Clardge MF, Dawah HA, Wilson MR A hierarchy of species concepts: The denouement in the saga of the species problem Species 1997 Chapman and Hall, London 381 424

[B22] De Queiroz K Howard J, Berlocher SH The general lineage concept of species Endless forms: Species and Speciation 1998 Oxford University Press, New York 57 75

[B23] De Queiroz K Ernst Mayr and the modern concept of species Proc Natl Ac Sci USA 2005 102 6600 6607 10.1073/pnas.0502030102 PMC1131873 15851674

[B24] Vos P Hogers R Bleeker M AFLP: a new technique for DNA fingerprinting Nucl Acids Res 1995 23 4407 4414 10.1093/nar/23.21.4407 7501463 PMC307397

[B25] Rosenberg NA Distruct: A program for the graphical display of population structure Mol Ecol Notes 2004 4 137 138 10.1046/j.1471-8286.2003.00566.x

[B26] Falush D Stephens M Pritchard JK Inference of population structure using multilocus genotype data: dominant markers and null alleles Mol Ecol Notes 2007 7 574 578 10.1111/j.1471-8286.2007.01758.x 18784791 PMC1974779

[B27] Evanno G Regnaut S Goudet J Detecting the number of clusters of individuals using the software structure: a simulation study Mol Ecol 2005 14 2611 2620 10.1111/j.1365-294X.2005.02553.x 15969739

[B28] Corander J Marttinen P Siren J Tang J Enhanced Bayesian modelling in BAPS software for learning genetic structures of populations BMC Bioinformatics 2008 9 539 10.1186/1471-2105-9-539 19087322 PMC2629778

[B29] Jing R Vershinin A Grzebyta J Shaw P Smýkal P Marshall D Ambrose MJ Ellis TH Flavell AJ The genetic diversity and evolution of field pea (*Pisum*) studied by high throughput retrotransposon based insertion polymorphism (RBIP) marker analysis BMC Evol Biol 2010 10 44 10.1186/1471-2148-10-44 20156342 PMC2834689

[B30] Vekemans X Beauwens T Lemaire M Roldán-Ruiz I Data from amplified fragment length polymorphism (AFLP) markers show indication of size homoplasy and of a relationship between degree of homoplasy and fragment size Mol Ecol 2002 11 139 151 10.1046/j.0962-1083.2001.01415.x 11903911

[B31] Zhivotovsky LA Estimating population structure in diploids with multilocus dominant DNA markers Mol Ecol 1999 8 907 913 10.1046/j.1365-294x.1999.00620.x 10434412

[B32] van den Berg RG Spooner DM A reexamination of infraspecific taxa of a wild potato, *Solanum microdontum *(*Solanum *sect. *Petota*: *Solanaceae*) Plant Syst Evol 1992 182 239 252 10.1007/BF00939190

[B33] Giannattasio RB Spooner DM A reexamination of species boundaries between *Solanum megistacrolobum *and *S. toralapanum *(*Solanum *sect. *Petota*, series *Megistacroloba*): morphological data Syst Bot 1994 19 89 105 10.2307/2419714

[B34] Giannattasio RB Spooner DM A reexamination of species boundaries and hypotheses of hybridization concerning *Solanum megistacrolobum *and *S. toralapanum *(*Solanum *sect. *Petota*, series *Megistacroloba*): Molecular data Syst Bot 1994 19 106 115 10.2307/2419715

[B35] Spooner DM Ugarte ML Skroch PW Species boundaries and interrelationships of two closely related sympatric diploid wild potato species, *Solanum astleyi *and *S. boliviense*, based on RAPDs Theor Appl Genet 1997 95 764 771 10.1007/s001220050623

[B36] Spooner DM Sytsma KJ Smith JF A molecular reexamination of diploid hybrid speciation of *Solanum raphanifolium* Evolution 1991 45 757 764 10.2307/2409926 28568823

[B37] Castillo RO Spooner DM Phylogenetic Relationships of Wild Potatoes, *Solanum *Series *Conicibaccata *(Sect. *Petota*) Syst Bot 1997 22 45 83 10.2307/2419677

[B38] Spooner DM Fajardo D Salas A Revision of the Solanum medians complex (Solanum section Petota) Syst Bot 2008 33 579 588 10.1600/036364408785679905

[B39] Hawkes JG Hjerting JP The potatoes of Bolivia: their breeding value and evolutionary relationships 1989 Oxford University Press, Oxford, UK

[B40] Spooner DM van den Berg RG An analysis of recent taxonomic concepts in wild potatoes (*Solanum *sect. *Petota*) Genet Res Crop Evol 1992 39 23 37 10.1007/BF00052651

[B41] Masuelli RW Camadro EL Erazzú LE Bedogni MC Marfil CF Homoploid hybridization in the origin and evolution of wild diploid potato species Plant Syst Evol 2009 277 143 151 10.1007/s00606-008-0116-x

[B42] Erazzú LE Camadro EL Clausen AM Persistence over time, overlapping distribution and molecular indications of interspecific hybridization in wild potato populations of Northwest Argentina Euphytica 2009 168 249 262

[B43] Schulte K Silvestro D Kiehlmann E Vesely S Novoa P Zizka G Detection of recent hybridization between sympatric Chilean *Puya *species (Bromeliaceae) using AFLP markers and reconstruction of complex relationships Mol Phylogenet Evol 2010 57 1105 1119 10.1016/j.ympev.2010.09.001 20832496

[B44] Alvarez NMB Peralta IE Salas A Spooner DM A morphological study of species boundaries of the wild potato Solanum brevicaule complex: replicated field trials in Peru Plant Syst Evol 2008 274 37 45 10.1007/s00606-008-0023-1

[B45] Ugent D The Potato Science 1970 170 1161 1166 10.1126/science.170.3963.1161 17744042

[B46] Spooner DM McLean K Ramsay G Waugh R Bryan GJ A single domestication for potato based on multilocus amplified fragment length polymorphism genotyping Proc Natl Acad Sci USA 2005 102 14694 16499 10.1073/pnas.0507400102 16203994 PMC1253605

[B47] Spooner DM van den Berg RG Species limits and hypotheses of hybridization of *Solanum berthaultii *Hawkes and *S. tarijense *Hawkes: morphological data Taxon 1992 41 685 700 10.2307/1222394

[B48] Spooner DM Fajardo D Bryan GJ Species limits of *Solanum berthaultii *Hawkes and *S. tarijense *Hawkes and the implications for species boundaries in Solanum sect. Petota Taxon 2007 56 987 999 10.2307/25065899

[B49] Ames M Salas A Spooner DM A morphometric study of species boundaries of the wild potato Solanum series Piurana (*Solanaceae*) and putatively related species from seven other series in Solanum sect. Petota Syst Bot 2008 33 566 578 10.1600/036364408785679789

[B50] Kardolus JP A biosystematic analysis of *Solanum acaule* PhD thesis 1998 Wageningen Agricultural University

[B51] Ames M Spooner DM Phylogeny of Solanum series Piurana and related species in Solanum section Petota based on five conserved ortholog sequences Taxon 2010 59 1091 1101

[B52] Hijmans RJ Spooner DM Geographic distribution of wild potato species Am J Bot 2001 88 2101 2112 10.2307/3558435 21669641

